# Technic of Stump Inversion in Appendectomy

**Published:** 1908-01

**Authors:** John Egerton Cannaday

**Affiliations:** Surgeon-in-Charge, Sheltering Arms Hospital, Hansford, W. Va.


### TECHNIC OF STUMP INVERSION IN APPENDECTOMY

BY JOHN EGERTON CANNADAY, M. D.

Surgeon-in-Charge, Sheltering Arms Hospital, Hansford, W. Va.

---

Authors Abstract from Journal American Medical Association.

---

Dr. Cannaday describes a modification of the usual method of inversion of the appendix stump. After the ligation and section of the mesoappendix a hemostat is first applied to the appendix at its junction with the cecum, a second hemostat is applied just above the first and slowly worked upward for about one quarter of an inch. The appendix is severed as near the first forcep as possible and the raw surface is touched with the thermocantory or tincture of iodine. The purse string suture of ten day chromic cat gut takes a dip into the tissue of the mesoappendix side so as to eliminate all possibility of hemorrhage this suture is passed over, not under the handle of the hemostat and around the other side of the appendix. When the loose ends of this suture are drawn taut, the handle of the hemostat is raised and the point depressed; this inverts the appendix easily and rapidly. There being no comparison in the use of the forcep in this way with the various appendix tuckers in use. The forcep being on throughout the operation prevents the possibility of any escape of fecal matter in the perineal cavity.

